# Assessment of level of asthma control and related factors in children attending pediatric respiratory clinics in Addis Ababa, Ethiopia

**DOI:** 10.1186/s12890-022-01865-8

**Published:** 2022-02-23

**Authors:** Amare Aschalew, Rahel Argaw Kebed, Takele Gezahegn Demie, Abate Yeshidinber Weldetsadik

**Affiliations:** 1 Bahir Dar University, Bahir Dar, Ethiopia; 2 Pediatric Pulmonary and Critical Care, Addis Ababa University, Addis Ababa, Ethiopia; 3 St. Paul’s Hospital Millennium Medical College, Addis Ababa, Ethiopia; 4 Pediatric Pulmonary and Critical Care, St. Paul’s Hospital Millennium Medical College, Addis Ababa, Ethiopia

**Keywords:** Asthma control, Child, Tertiary care centers, Ethiopia

## Abstract

**Background:**

Asthma is a common airways disease with significant morbidity and mortality in all ages. Studies of pediatric asthma control and its determinants yielded variable results across settings. However, there is paucity of data on asthma control and its factors in Ethiopian children. We aimed to assess the level of asthma control and the related factors in children attending pediatric respiratory clinics at three tertiary hospitals in Addis Ababa.

**Methods:**

We conducted a cross-sectional study from March 1 to August 30, 2020 using standardized questionnaires and review of patient’s charts. Data was analyzed using SPSS software for window version 26.

**Results:**

A total of 105 children (56.2% male) were included in the study. The mean age (± SD) and age at Asthma diagnosis (± SD) were 6 (± 3.3) and 4 (± 2.8) respectively. Uncontrolled asthma was present in 33 (31%) of children. Comorbidities (Atopic dermatitis and allergic Rhinitis (AOR = 4.56; 95% CI 1.1–18.70; *P* = 0.035), poor adherence to controller medications (AOR = 3.23; 95% CI 1.20–10.20; *P* = 0.045), inappropriate inhaler technique (AOR = 3.48; 95% CI 1.18–10.3; *P* = 0.024), and lack of specialized care (AOR = 4.72; 95% CI 1.13–19.80; *P* = 0.034) were significantly associated with suboptimal asthma control.

**Conclusion:**

One-third of children attending pediatric respiratory clinics in Addis Ababa had uncontrolled Asthma. Treatment of comorbidities, training of appropriate inhaler techniques, optimal adherence to controllers, and proper organization of clinics should be emphasized to improve asthma control among children.

## Introduction

Asthma is among the most common respiratory diseases with significant morbidity and mortality across all ages [1]. The World Health Organization (WHO) estimates 339 million people suffer from asthma worldwide [1, 2]. Asthma is among the most common pediatric diseases in African children with a prevalence range of 9% in Ethiopia to 20% in South Africa [3]. A recent review showed that overall prevalence of asthma was 13.9% (95% CI 9.6–18.3) in African children younger than 15 years [4]. In 2015, Asthma was estimated to cause 14.7 deaths per 100,000 individuals in Ethiopia, ranking 17th among the top 20 causes of mortality [5].

The major goal of asthma management is to achieve control of the disease and to improve the quality of life of affected children and their families, avoid school absenteeism, increase productivity, and prevent the need for emergency care and hospitalizations [6–9]. However, most patients, especially children, do not receive optimal asthma management secondary to poor inhaler technique and other complex factors [10, 11]. Multiple previous studies have documented variable but important results of pediatric asthma control and its factors in different settings. Prevalence of poorly controlled asthma in these pediatric studies varied from as low as 16% to as high as 84% [12–19]. Major factors related to poor asthma control, as documented in previous studies include, but not limited to, respiratory tract infections, lack of shared decision-making, inadequate asthma education, poor drug adherence, asthma severity, presence of comorbidities and other sociodemographic factors, previous asthma exacerbation, and type of controller medication [10, 20–28].

Asthma control and its determinants vary from practice setting as environmental, socio-demographic, medication adherence and availability, and household factors differ significantly across settings [13, 14, 29]. Previous work in Ethiopia, showed that in a tertiary hospital in Addis Ababa a third of adult asthmatics had poor asthma control [30]. However, there are limited data on childhood asthma control from low-income countries including Ethiopia. Thus, this study aimed to assess the level of and factors related to asthma control in children attending pediatric respiratory clinics in three tertiary centers in Addis Ababa, Ethiopia. Knowledge of level of asthma control and related factors will help to develop strategies to improve asthma care and decrease asthma morbidity and mortality in Ethiopia and potentially other low-income countries.

In this study, we defined bronchial asthma as physician-diagnosed asthma consistent with asthma symptoms and signs (episodic wheezing, cough, shortness of breath, chest tightness), and airway reversibility evidenced by response to short acting beta agonists and/or spirometry (when available) in children older than 5 years of age. Preschool asthma was defined as frequent (≥ 8 days/month) asthma-like symptoms or recurrent (≥ 2) exacerbations (episodes with asthma-like signs) in children 1–5 years old as defined by the Canadian pediatrics society and response to asthma medications [31].

## Methods and materials

### Study design and settings

The study was conducted in Addis Ababa, Ethiopia. The Ethiopian health system follows a primary, secondary and tertiary level of referrals, and patients mainly pay out of pocket for health care services including drugs while government pays for those who didn’t afford to pay.

A hospital-based cross-sectional study was conducted from March 1 to August 30, 2020 in 3 tertiary teaching hospitals (Tikur Anbessa Specialized Hospital (TASH), St. Paul’s Hospital Millennium Medical College (SPHMMC), and Yekatit 12 Hospital Medical College (Yekatit 12)) in Addis Ababa, Ethiopia. The hospitals were selected as they have respiratory clinics providing clinical care for children with asthma.

TASH is the largest university hospital in the country. The pediatric respiratory clinic at TASH is open twice weekly and is led by a pediatric pulmonologist and consists of a team of clinicians including a pediatric pulmonologist, pediatric pulmonology fellow, senior pediatric residents rotating to the clinic, and nursing staff. An average of 60 patients with respiratory diseases are evaluated in the pulmonary clinic per month.

SPHMMC is the second largest hospital in Ethiopia. There is a once weekly follow up at the pediatric respiratory clinic for pediatric pulmonary diseases including asthma. Skin allergy test and spirometry services are available regularly in SPHMMC while the other two hospitals lack these services. The respiratory clinic is led by a pediatric pulmonologist and consists of a team of clinicians including a pediatric pulmonology fellow, and senior pediatric residents in their pulmonology rotation.

Yekatit 12 is a teaching hospital in Addis Ababa and its pediatric respiratory clinic is open once weekly providing service by a team consisting of a pediatrician, residents, and nursing staff but no pediatric pulmonologist. The asthma care is mostly handled by residents with inconsistent availability of pediatricians and care less structured compared to the other two centers where the clinics are organized and led by pediatric pulmonologists.

### Participants and sampling method

Children 1–14 years old with physician‑diagnosed asthma, who visited the 3 pediatric respiratory clinics, and are on controller therapy for a minimum of 3 months were included. Patients with cardiorespiratory comorbidities and those with uncertain diagnoses were excluded despite being on controller medications.

Sample size was calculated using single population proportion formula with a 95% confidence interval, 5% margin of error and prevalence of 18% from a previous Nigerian study. After correction for a finite population, the final sample size was 107. However, only 105 (98% of the calculated sample size) patients fulfilled the inclusion criteria. Among them, 39 (37%) children were from TASH, 42 (40%) from SPHMMC whereas 24 (23%) were from Yekatit 12 hospital.

### Data collection and measurements

Trained general practitioners and pediatric residents collected the data by interview of the child and /care-giver using a pre-tested standardized questionnaires and check lists. Asthma control was evaluated by using standardized age appropriate tools; Test for Respiratory and Asthma Control in Kids (TRACK) for < 5 years [32], Childhood-Asthma control test (C-ACT) for 5–< 12 years [33], and Asthma Control Test (ACT) for ≥ 12 years [34]. Asthma Control Test (ACT) is a 5-point questionnaire used to assess asthma control in children 12–14 years of age.

The Childhood Asthma Control Test (C-ACT) is a 7-point questionnaire used to assess asthma control in children 6–12 years of age, while Test for Respiratory and Asthma Control in Kids (TRACK) is a 5-point questionnaire used to assess asthma control for children under five years old. Uncontrolled asthma was defined based on a total C-ACT or ACT score ≤ 19 or TRACK score ≤ 80 as appropriate for age and drug adherence was considered optimal if medication adherence is greater than 80% as determined by medications adherence response scale for asthma (MARS-A score) [35, 36].

### Data processing and analysis

Data was analyzed using SPSS version 26 after cleaning before analysis. Descriptive statistics like frequencies, mean, and standard deviations (SDs) were used to summarize the independent variables. Chi- square test with bivariate analysis were performed to evaluate the association between dependent and independent variables. Odds ratios (ORs) with 95% confidence interval (CI) were estimated to see strength of the associations and *p*-value ≤ 0.05 was considered to be of statistical significance. Missing values were handled by subgroup analysis.

## Results

### Sociodemographic character of study subjects

The study included 105 children with asthma of which 59 (56.2%) were males. Mean (± SD) current age and age at asthma diagnosis (± SD) were 6 (± 3.3) and 4 (± 2.8) years respectively. More than 71% of children were less than 12 years of age (Table 1). A third of caregivers were housewives (33%) and educated either to primary (6.7%) or secondary (45.7%) level. Most (83%) children were from Addis Ababa. Family history of asthma was present in only 21 (20%) children with a 12% rate of parental asthma history (Table 2).

**Table 1:** Sociodemographic character of children with asthma in teaching hospitals, Ethiopia, March 2020 (N = 105)

Variables	Controlled (n = 72), n (%)	Uncontrolled (n = 33), n (%)	*P*-value
Gender			
Male	41 (57)	18 (54.5)	0.818
Female	31 (43)	15 (45.6)	
Age (in years)			
1–5	33 (46)	19 (58)	0.682
6–12	36 (50)	11 (33)	
> 12	3 (4)	3 (9)	
Mean ± SD			
Address			
Urban	60 (83)	27 (82)	0.848
Rural	12 (17)	6 (18)	
Caregiver occupation			
Unemployed	15 (21)	4 (12)	NS
Employed	35 (48.6)	16 (48.5)	NS
Housewives	22 (30.4)	13 (39.5)	NS
Caregiver’s level of education			
Primary or less	32 (44.4)	18 (54.5)	NS
Secondary and beyond	40 (55.6)	15 (45.5)	NS
Family size			
3–5	42 (58)	15 (45.5)	0.145*
More than five	30 (42)	18 (54.5)	
EBF	55 (76)	28 (24)	0.327
Gestational age			
Term	67 (93)	27 (82)	0.092*
Preterm	5 (7)	6 (18)	

EBF, exclusive breastfeeding; SD, standard deviation; NS, not significant

(NB. *Variables with *P*-values less than 0.2 included in multivariate analysis).

**Table 2:** Comorbid conditions and trigger factors related to Asthma control among children at three teaching hospitals in Addis Ababa, Ethiopia (N = 105)

Variables	Controlled (n = 72), n (%)	Uncontrolled (n = 33), (%)	*P*-value
Previous asthma related admission	64 (66)	33 (34)	0.999
Family history of asthma			
Yes	14 (66.7)	7 (33.3)	0.417
No	58 (69)	26 (31)	
Household smoke exposure	29 (66)	15 (34)	0.618
Comorbid conditions			
Allergic rhinitis only	9 (90)	1 (10)	0.326
Atopic dermatitis only	10 (59)	7 (41)	0.184*
Allergic rhinitis and atopic dermatitis	7 (41)	10 (59)	0.01*
No comorbid factors	46 (75)	15 (25)	0.184*
Total	45 (42.8%)		
Trigger factors			
URTI	7 (70)	3 (30)	1.000
Dust/Fumes	1 (100)	0 (0)	1.000
Cold weather	6 (86)	1 (14)	1.000
URTI and/cold weather/dust	57 (66)	29 (34)	1.000
Cigarette smoke	0	2 (100)	
Unknown triggers	1 (50)	1 (50)	1.000
Total	86 (83%)		

Figures in parentheses are the percentages of total in each row (NB. *Variable with *P*-values less than 0.2, included in Multivariate analysis)

### Asthma severity, control and related factors

While the overall level of asthma control in our study was 69%, there was a difference between the 3 hospitals with a rate of 79% in SPHMMC, 72% in TASH, and 46% in Yekatit 12 Hospital. There was no association between child’s gender, age, family size, gestational age, type of controller medication, caregivers’ level of education and level of asthma control in the child (Tables 3, 4). Most children (60 (57%) had mild persistent asthma followed by moderate persistent asthma in 39 (37%) and most were on GINA step 2 management (Table 5). Pertaining to objective diagnosis of asthma, only 20 (19%) children had spirometry supported diagnosis of asthma because of age limitation for most children, and test unavailability for some of them.

**Table 3:** Drug adherence, inhaler technique, and place of care related to Asthma control in Addis Ababa, Ethiopia (n = 105)

Variables	Controlled (n = 72), n (%)	Uncontrolled (n = 33), n (%)^+^	Total (N = 105)	*P*-value
Drug adherence				
Yes	54 (79.4)	14 (20.6)	68	0.002*
No	18 (48.6)	19 (51.4)	37	
Inhaler techniques				
Appropriate	35 (95)	2 (5)	37	
Inappropriate	21 (47)	24 (53)	45	< 0.001*
Follow up hospital				
SPHMMC	33 (79)	9 (21)	42	0.492
TASH	28 (72)	11 (28)	39	0.481
Yekatit 12	11 (46)	13 (54)	24	0.008*

23 patients in the Montelukast group excluded for inhaler technique assessment

^+^n indicates frequency

^%^Stands for percentages

^*^Variable with *p*-values less than 0.2 included in multivariate analysis

**Table 4:** Binary multivariable regression for predictors of uncontrolled pediatric asthma in three hospitals in Addis Ababa, Ethiopia, March 2020 (N = 105)

Variable	Response categories	Level of Asthma control		COR (95% CI)	AOR (95% CI)	*p*-value
		Controlled	Uncontrolled			
Presence comorbidities	No comorbidity	46 (75%)	15 (25%)	1.000	1.000	
	Allergic Rhinitis	9 (90%)	1 (10%)	0.34 (0.04–3)	0.494 (0.042–5.9)	0.578
	Atopic dermatitis	10 (59%)	7 (41%)	2.1 (0.69–6.6)	2.597 (0.576–11.7)	0.214
	Both	7 (41%)	10 (59%)	4.4 (1.4–13.5)	4.56 (1.1–18.7)	0.035
Controller adherence	Yes	54 (79.4%)	14 (20.6%)	1.000	1.000	
	No	18 (48.6%)	19 (51.4%)	0.25 (0.1–0.59)	3.23 (1.2–10.2)	0.045
Appropriate Inhaler technique	Yes	35 (95%)	2 (5%)	1.000	1.000	
	No	21 (47%)	24 (53%)	5.2 (2.13–12.80)	3.48 (1.18–10.3)	0.024
Place of care	SPHMMC	33 (79%)	9 (21%)	1.000	1.000	
	TASH	28 (78%)	11 (28%)	1.4 (0.5–3.9)	1.37 (0.37–5.0)	0.636
	Yekatit 12	11 (46%)	13 (54%)	4.3 (1.5–12.9)	4.72 (1.13–19.8)	0.034

Note that only comorbidities, inhalation technique, adherence and place of care are associated with asthma control

Other covariates including age, sex, family size, care givers level of education and occupation were included in the model but were not related to level of asthma control

**Table 5:** Controller treatment of children with asthma in Addis Ababa, (N = 105)

Controller type		Frequency	Percent
	Inhalational Steroid	75	71.4
	Monetlukuast	23	21.9
	Stepped down and off*	7	6.7
	Total	105	100.0

*Controllers discontinued recently (with in the last 3 months) because of good asthma control after stepping down and currently not requiring controller

### Comorbid conditions and triggers related to asthma control

Common comorbidities were atopic dermatitis and allergic rhinitis documented in 42.8% of children. Specific trigger factors for asthma exacerbation and control were identified in 86 (83%) children. The most common triggers for exacerbation were combinations of upper respiratory tract infections (URTI) and Cold weather (55%), URTI and cold weather with dust (26.4%) (Table 2). Most of the children had been hospitalized for asthma exacerbation at least once in the past with 72% hospitalized twice or more for asthma exacerbations (Table 2).

Children with both allergic rhinitis and atopic dermatitis were more likely to have uncontrolled asthma (AOR = 4.56; 95% CI 1.11–18.70; *P* = 0.035). However, there was no significant association between triggering factors, previous asthma related admissions, household or tobacco smoke exposures and asthma control. Likewise, family history of asthma and type of fuel used for cooking were not associated with the level of asthma control (Table 2).

Less than 50% of households used clean fuel for cooking; the rest used electricity with unclean fuel (kerosene and firewood) for cooking (55%). Only 2 (1.9%) children were exposed to cigarettes smoke at home.

### Drug adherence, inhaler technique, and place of care

Two-third of patients were adherent to their medications, and most of them had controlled asthma (79.4%). The reasons for non-adherence were forgetting to give/take controllers, not knowing how much to give/take, and inadequate prescriptions prior to the next appointment. Inhaler technique was inappropriate in 45 (55%) of children. Beclomethasone was the most commonly used controller in 75 (71%) children followed by Montelukast in 23 (22%). The rest were stepped-down and stopped their controllers because their asthma was well controlled for an adequate time. No patient was followed with written asthma action plan.

Children who were not adherent to medications were 3 times more likely to have uncontrolled Asthma (AOR = 3.23; 95% CI 1.2–10.2; *P* = 0.045)**.** Likewise, children who had poor inhaler techniques were 3.5 times more likely to have uncontrolled asthma (AOR = 3.48; 95% CI 1.19–10.3; *P* = 0.024). Asthma control was significantly lower in children from Yekatit-12 hospital (AOR = 4.723; 95% CI 1.125–19.818; *P* = 0.034). However, there was no association between asthma control and the type of controller used.

### Factors associated with uncontrolled asthma

A number of independent predictors of asthma control were found on multivariate analysis (Table 4). The presence of comorbid conditions (atopic dermatitis and allergic rhinitis), poor inhaler technique, poor adherence to controller drugs, and the specific health care facility independently predicted uncontrolled asthma among the study participants.

## Discussion

Our study assessed the level of asthma control and related factors in children attending pediatric respiratory clinics at three tertiary hospitals in Addis Ababa, Ethiopia. Our study demonstrated that every 1 in 3 asthmatic children had an uncontrolled asthma. As current asthma management primarily aims optimal asthma control, it is critical to understand the burden and predictors of uncontrolled asthma in children [6, 7].

Our rate of uncontrolled asthma was lower compared to reports from USA (46%), and Saudi Arabia (59–84%) [19, 24, 26]. This may be due to differences in environmental factors [12, 13, 15] as well as sociodemographic and practice factors including better parental understanding, health seeking and reporting culture from parents and children in middle- and high-income settings [14]. Additionally, the use of different assessment tools, inclusion of different age groups and disease severity may lead to discrepancies of asthma control results. On the contrary, our study showed lower asthma control compared to a single center Nigerian study (18%) [23]. It is possible that at least some of our children are undertreated for their asthma contributing to our poor asthma control. On the other hand, the difference may also be related to the multicenter nature of our study with very low asthma control in one of our centers (Yekatit 12) with uncontrolled asthma of only 21% in a different center (SPHMMC), comparable to the Nigerian study. Additionally, limited follow up of patients with controlled asthma at follow up clinics due to the COVID-19 might have contributed to higher rate of uncontrolled asthma in our children. The other possible reason might be shortage and irregular availability of controller medications especially beclomethasone in the preceding 2 years of the study.

Similar to our work, previous studies reported comorbid allergic conditions to affect asthma control significantly [21, 24, 26]. Saudi Arabian [17] and Australian [21] studies also documented poor drug adherence and inappropriate inhaler techniques to significantly increase uncontrolled Asthma in children.

We observed that the presence of both allergic rhinitis and dermatitis predicted asthma control but neither of them do separately. While presence of allergy may not consistently predict asthma control [37, 38], asthma with both allergic rhinitis and dermatitis (‘atopic march’) marks a special group of children with different genetic and immunologic state possibly contributing to variable severity and asthma control [37, 39]. However, our result should be interpreted with caution as the small sample size and under reporting of allergic rhinitis and dermatitis might have affected our result.

Children followed at Yekatit 12 had lower asthma control compared to the other two centers. This may be related to differences in care providers/physicians and alternative structuring of the clinic compared to the other two centers. A previous study documented better rate of asthma control cared by a specialist (26% uncontrolled) compared to non-specialists (49% uncontrolled) [40].

The type of controller medication, previous asthma exacerbation, and caregivers’ level of education were documented to affect asthma control in previous studies [20, 26, 27], but were not found to be significant in our study. However, our result should be interpreted cautiously as our sample size and similarity of educational and sociodemographic status in controlled and uncontrolled asthmatics may affect the result.

Additionally, contrary to some previous studies [22–25], asthma severity, rural/urban residence, and family income were not found to affect asthma control in our children. However, we acknowledge that our result has limited generalizability for children from rural area and asthmatics with comorbidities other than allergic rhinitis and dermatitis.

Despite the multicenter nature, our study is limited by relatively small sample size due to the COVID-19 pandemic overlapping with our study period. Similarly, our level of Asthma control might be affected by selection bias as more children with poor asthma control are likely to be available while mild and controlled asthma cases may not attend the follow-up clinics during such unprecedented period. Additionally, few patients were followed up only through phone calls and were not attending the clinics. Additionally, our asthma diagnosis was supplemented by spirometry only in a fifth of children, and the higher proportion of preschool asthmatics puts our asthma diagnosis less precise despite our pragmatic approach as used in larger studies. As a result of this the generalizability of our work may be limited.

In conclusion, one-third of children under follow up in public pediatric centers in Addis Ababa had uncontrolled asthma. Comorbidities (allergic rhinitis and atopic dermatitis), poor adherence to controllers, facility of care, and inappropriate inhaler techniques are important predictors of uncontrolled Asthma. Treatment of comorbidities, proper education and training of inhaler technique and controller adherence, and organizing respiratory clinics preferably with dedicated pediatricians/pulmonologist when available should be emphasized to optimize the main goal of optimal asthma care in Ethiopia.

## Data Availability

Data is available with the authors for review based on request from the corresponding author.

## Abbreviations

ACT: Asthma Control Test
C-ACT: Childhood Asthma Control Test
COVID-19: SARS-Cov-2 caused Corona Virus disease
DALY: Disability adjusted life years
GINA: Global initiative for asthma
ICS: Inhaled corticosteroid
IRB: Institutional Review Board
ISAAC: International Study of Asthma and Allergy in Children
MARS-A: Medication adherence response scale for Asthma
OCS: Oral Corticosteroids
OPD: Out Patient Department
RCT: Randomized control trial
SABA: Short Acting Beta Agonists
SPHMMC: St. Paul’s Hospital Millennium College
TASH: Tikur Anbesa Specialized Hospital
TRACK: Test for Respiratory and Asthma Control in Kids
WHO: World Health Organization
